# Novel Sources of Witchweed (*Striga*) Resistance from Wild Sorghum Accessions

**DOI:** 10.3389/fpls.2017.00116

**Published:** 2017-02-06

**Authors:** Dorothy A. Mbuvi, Clet W. Masiga, Eric Kuria, Joel Masanga, Mark Wamalwa, Abdallah Mohamed, Damaris A. Odeny, Nada Hamza, Michael P. Timko, Steven Runo

**Affiliations:** ^1^Department of Biochemistry and Biotechnology, Kenyatta UniversityNairobi, Kenya; ^2^Tropical Institute of Development InnovationsKampala, Uganda; ^3^Sudan Academy of SciencesKhartoum, Sudan; ^4^Agricultural Research CooperationWad-Medani, Sudan; ^5^International Crops Research Institute for the Semi-Arid TropicsNairobi, Kenya; ^6^Commission for Biotechnology and Genetic Engineering, National Centre for ResearchKhartoum, Sudan; ^7^Department of Biology, University of VirginiaCharlottesville, VA, USA

**Keywords:** *Striga* resistance, witchweed, sorghum, wild sorghum relatives, Sub-Saharan Africa, *Striga* hermonthica benth., witchweed, sorghum bicolor

## Abstract

Sorghum is a major food staple in sub-Saharan Africa (SSA), but its production is constrained by the parasitic plant *Striga* that attaches to the roots of many cereals crops and causes severe stunting and loss of yield. Away from cultivated farmland, wild sorghum accessions grow as weedy plants and have shown remarkable immunity to *Striga*. We sought to determine the extent of the resistance to *Striga* in wild sorghum plants. Our screening strategy involved controlled laboratory assays of rhizotrons, where we artificially infected sorghum with *Striga*, as well as field experiments at three sites, where we grew sorghum with a natural *Striga* infestation. We tested the resistance response of seven accessions of wild sorghum of the aethiopicum, drummondii, and arundinaceum races against N13, which is a cultivated *Striga* resistant landrace. The susceptible control was farmer-preferred variety, Ochuti. From the laboratory experiments, we found three wild sorghum accessions (WSA-1, WSE-1, and WSA-2) that had significantly higher resistance than N13. These accessions had the lowest *Striga* biomass and the fewest and smallest *Striga* attached to them. Further microscopic and histological analysis of attached *Striga* haustorium showed that wild sorghum accessions hindered the ingression of *Striga* haustorium into the host endodermis. In one of the resistant accessions (WSE-1), host and parasite interaction led to the accumulation of large amounts of secondary metabolites that formed a dark coloration at the interphase. Field experiments confirmed the laboratory screening experiments in that these same accessions were found to have resistance against *Striga*. In the field, wild sorghum had low Area under the *Striga* Number Progressive curve (AUSNPC), which measures emergence of *Striga* from a host over time. We concluded that wild sorghum accessions are an important reservoir for *Striga* resistance that could be used to expand the genetic basis of cultivated sorghum for resistance to the parasite.

## Introduction

Domestication—the process of transforming wild species into elite cultivars—inevitably leads to decreased genetic diversity in the selected crops (Doebley et al., 2006). In some cases, the lost genetic diversity may represent the organism's capacity to adopt changes, such as pathogen resistance, in a dynamic ecosystem (Sakai and Itoh, 2010). Based on this possibility, many crop improvement programmes are now using genomics and molecular genetic technologies to reclaim lost genetic diversity by specifically targeting genes responsible for pathogen resistance (Zhu et al., 2012; Jones et al., 2014). Success of these programmes is based on well-documented evidence that wild relatives of crops are useful reservoirs of disease resistance genes (Brozynska et al., 2016). In this study, we explore the resistance interactions between wild sorghum accessions and the parasitic plant *Striga hermonthica* at their the parasite's center of origin in northeastern Africa.

*Striga* is also infamously known as witchweed and can destroy a crop with up to a 100% yield loss (Ejeta, 2007). It is estimated that over 60% of farmland under cultivation in sub-Saharan Africa (SSA) is infested with one or more species of *Striga*, which impacts over 300 million farmers in over 25 countries with yield losses of over seven billion dollars (Ejeta, 2007). Three species of *Striga* are particularly destructive—*S. hermonthica* (Del.) Benth. and *S. asiatica* (L) Kuntze, which attack cereals, and *S. gesnerioides* (Willd.) Vatke, which is parasitic to cowpea plants.

Such great losses indicate a successful parasite, and for *Striga*, this success can be attributed to two aspects of its lifecycle: elevated fecundity, each *Striga* flower spike can produce over 50,000 seeds that remain viable in the soil for up to 14 years (Yoder and Scholes, 2010), and its remarkable ability to intimately link its life cycle to that of a host, such as when germination of *Striga* seeds and attachment to the host only occur in response to chemical cues (strigolatone) contained in the host and in some cases, non-host root exudates (Bouwmeester et al., 2007).

To manage *Striga*, smallholder farmers in SSA have fought back with different control methods aimed at reducing *Striga* seed density in the soil. These methods include reducing the amount of *Striga*-seed-contaminated crop seed supplies, hand weeding, crop rotation, and the use of “trap crops” that induce germination of the parasite but are not hosts. Although these methods have been extensively encouraged for many years, crop losses and the host range of *Striga* has continued to increase, which underscores the need for a sustainable *Striga* management strategy (Cotter et al., 2012). The ideal strategy would be an integrated approach that greatly exploits natural resistance. However, sources of *Striga* resistance are limited and are often overcome by the parasite (Ejeta, 2007). Therefore, additional sources of *Striga* resistance need to be found for introduction to farming systems and to promote long-term resistance.

We hypothesized that *Striga*-resistant sorghum was likely to be found in northeastern Africa since this region harbors the greatest diversity of both wild and cultivated sorghum (Paterson et al., 2013) and because this area is the natural range of the *Striga* parasite (Musselman and Hepper, 1986). Sorghum in northeastern Africa is highly variable and complex, but most genotypes can be classified as wild, cultivated, or cultivated-wild crossbreeds—all species can be classified as subspecies that are completely inter-fertile (Harlan and de Wet, 1972). On one hand, cultivated types are classified as subsp. *bicolor* and further subspecies are classified into five different races based on grain shape, glume shape, and panicle type (Harlan and de Wet, 1972). The five basic races are bicolor, durra, kafir, caudatum, and guinea (Paterson et al., 2013). On the other hand, wild sorghums are classified as subsp. *Verticilliflorum*, which consists of the races arundinaceum, virgatum, aethiopicum, and verticilliflorum (Harlan and de Wet, 1972). In addition, crossbreeds between bicolor and wild sorghum occur and are classified as drummondii (Paterson et al., 2013).

Domesticated sorghum genotypes are to a great extent susceptible to *Striga* (Ejeta, 2007). In contrast, wild sorghum can be found on uncultivated land as weeds that are immune to *Striga* infestation, which suggests there is potential for wild sorghum to be used a source of *Striga* resistance.

Indeed, for cultivated sorghum, only a few races are known to harbor *Striga* resistance. Among these is landrace N13 (*S. bicolor* subspecies *bicolor* race durra), which has a resistance mechanism that was described in detail by Maiti et al. (1984). N13 has been known to resist *Striga* by cell wall-thickening as well as depositing silica. In addition, *Striga* can induce extra lignification in the pericycle cells to the point that the haustorium encounters the endodermis. The haustorium then becomes weak and is limited by xylem-xylem connections with the host (Maiti et al., 1984).

In this study, N13 was used as a resistance control alongside a susceptible control—Ochuti classified as *S. bicolor*, subspecies *bicolor*. Ochuti is a sorghum variety preferred by farmers and is a popular crop in Western Kenya (Ngugi et al., 2016).

With regard to the resistance of wild sorghum to *Striga*, several studies have previously described low germination stimulant production, germination inhibition, and low haustorial initiation production as a form of resistance (Rich et al., 2004) as well as hypersensitive reaction (HR) resistance against *S. asiatica* (Mohamed et al., 2003). Furthermore, HR has been observed as an incompatible response between the hypervirulent *S. gesnerioides* race SG3 and resistant b301 cowpea variety (Li and Timko, 2009).

There is another possible mechanism of resistance against *Striga*—physiological barriers could lead to deposits of material that obstruct haustorium penetration. This mechanism was demonstrated in resistance against *S. gesnerioides* (Okonkwo and Nwoke, 1978). Substances that are stained darkly by toluidine blue characterize this mechanism of resistance (Maiti et al., 1984). Although this resistance was not well characterized, it is believed that these substances soften and/or dissolve the cell wall of host tissues (Rogers and Nelson, 1962). Aside from enzymes that degrade host tissues, there are secondary metabolites whose role in defending hosts against pathogens is becoming apparent (van Dam and Bouwmeester, 2016). Although not characterized well in parasitic plants, their role in acquiring hosts is undeniable.

The wild sorghum accessions described in this paper include the *S. bicolor* subspecies verticilliflorum and the races arundinaceum, aethiopicum, and drummondii. These are part of a large collection maintained at the Agricultural Research Corporation (ARC) in Sudan. The collections were carried out in *Striga*-prone areas of Sudan.

To determine the resistance response of these wild sorghum accessions and their potential to function as donors of *Striga* resistance, we used laboratory and field screening assays. We showed remarkable *Striga* resistance in wild sorghum accessions of aethiopicum and arundinaceum races. We found this resistance was mediated by possible mechanical or biochemical barrier mechanisms. Our study thus provides the potential to increase the genetic basis of cultivated sorghum. These findings will have wide-reaching implications for *Striga* control because of the ability to pyramid (i.e., combine) multiple genes in a single variety so that resistance is durable and covers a broad spectrum.

## Materials and methods

### Plant materials

Before this study was conducted, the Agricultural Research Cooperation (ARC) in Sudan conducted a countrywide collection of wild sorghum accessions and maintained them at a research station. The collection consisted of cultivated sorghum as well as wild sorghum. Genotypes collected from different locations were treated as different accessions. For this study, we selected a wild sorghum germplasm comprised of three wild races: aethiopicum (1 accession), arundinaceum (3 accessions), and drummondii (3 accessions). These accessions were referred to as WSE-1, WSA-1, WSA-2, WSA-3, WSD-1, WSD-2, and WSD-3, respectively. The initials “W,” “S,” “E,” “A,” and “D” denote Wild, Sudan, aEthiopicum, Arundinaceum, and Drummondii. The *Striga*-resistant landrace N13 was used as a resistant control for both laboratory and field experiments. In addition, Ochuti, which is sorghum cultivar that is popular among farmers in Kenya, was used for the susceptibility check. Both N13 and Ochuti were part of a sorghum collection maintained at Kenyatta University and originally obtained from the International Crops Research Institute for the Semi-Arid Tropics (ICRISAT) in Nairobi. *S. hermonthica* seeds were collected from sorghum growing in *Striga*-infested fields in Western Kenya at Kibos, Mbita, and Alupe.

### Preconditioning of *Striga* seeds

Prior to germination, *Striga* seeds were preconditioned as described in Gurney et al. (2003). Seeds (25 mg) were first surface sterilized in 10% (v/v) commercial bleach for 10 min with gentle agitation. The seeds were then rinsed three times with double distilled water and spread on a glass fiber filter paper (Whatman GFA) placed on sterile petri dishes. Sterile distilled water (5 ml) was added to *Striga* seeds followed by incubation at 29°C for 11 days for pre-conditioning. Preconditioned seeds were germinated by adding 3 ml of 0.1 ppm GR24 and incubated overnight at 29°C. Germinated *Striga* seedlings were analyzed for germination efficiency under a Leica MZ7F stereomicroscope fitted with a DFC320FX camera (Leica UK), and only plates showing >70% germination were used to infect sorghum roots.

### Infection of sorghum roots with *Striga*

Sorghum seeds were germinated between moistened blocks of cotton wool lined with filter paper. After 7 days, each sorghum seedling was transferred to a root observation chamber (rhizotron)—25 × 25 × 5 cm Perspex plate—packed with vermiculite, as described by Gurney et al. (2006). The rhizotrons were covered with aluminum foil and supplied with 25 ml of 40% Long Ashton nutrient solution (Hudson, 1967) twice a day. The plants were maintained in a glasshouse for 11 days in a 12-h photoperiod. Day and night temperatures were set at 28 and 24°C, respectively, and the relative humidity was set at 60%.

After 11 days, sorghum seedlings with well-developed roots were infected with 25 mg of pre-germinated *S. hermonthica* seedlings by aligning them on sorghum roots using a soft paintbrush. Three different ecotypes of *Striga* (Kibos, Mbita, and Alupe) were used for infection. Five sorghum plants per accession were screened, and the experiment was replicated three times.

### Macroscopic screening of sorghum for *Striga* resistance

Rhizotrons containing sorghum roots infected with *Striga* were observed for resistance at 3, 9, and 21 days after infection (DAI). Observations at 3 and 9 DAI were done using a Leica MZ7F stereomicroscope fitted with a DFC320FX camera (Leica UK). At 21 DAI, rhizotrons were photographed using a Canon EOS600D camera.

To screen sorghum for post-attachment resistance and the effects of host plants on parasite development, *Striga* plants were harvested from the infected roots at 21 DAI. Harvested *Striga* seedlings from each host plant were placed in a 90 mm Petri dish and photographed using a digital camera. The number and length of *Striga* seedlings parasitizing each host plant was determined from the photographs using the image analysis software ImageJ, v. 1.45 (http://rsb.info.nih.gov/ij/). In addition, total *Striga* biomass was determined after extracting all *Striga* seedlings parasitizing each sorghum and drying the seedlings at 45°C for 2 days. The same metrics—*Striga* length, number, and biomass—were used to deduce the virulence of *Striga* ecotypes.

### Microscopic screening of *Striga* resistance

To determine the extent of parasite development within the host root cortex, root tissue at the point of *Striga* haustoria attachment was dissected from host plants at 3 and 9 days (DAI) for sectioning. We used Technovit 7100 (embedding) and Technovit 3040 mounting kits (Haraeus Kulzer GmbH). The samples were fixed using Carnoy's fixative (4:1, ethanol:acetic acid) then dehydrated twice in 100% ethanol for 30 min. Next, the samples were pre-infiltrated in Ethanol-Technovit solution (1:1) for 1 h then in 100% Technovit solution for an additional 1 h. Fresh Technovit solution was added, and the samples were incubated for 3 days at 4°C.

To embed the tissues, the samples were placed into Eppendorf (1.5 ml) tube lid molds and Technovit solution:hardener 1 (1:15) was added. The molds were mounted onto wooden blocks using the Technovit 3040 kit according to the manufacturer's instructions. For sectioning, 5 micron-thick slices were cut using a Leica RM 2155 microtome (Leica instruments GmbH) and transferred to microscope slides. The sections were stained using 0.1% toluidine blue O (Sigma, USA) in 100 mM phosphate buffer at pH 7 for 2 min, then washed in distilled water and dried at 65°C for 30 min on a hot plate. The sections were then mounted onto glass slides with DePex (BDH, Poole, UK) then observed and photographed using a Zeiss microscope mounted with a Canon camera.

### Field-site screening of sorghum accessions for *Striga* resistance

The field evaluation of *Striga* resistance reported in this paper was part of a larger study involving 107 sorghum accessions that was conducted for a genome-wide association-mapping project. A sketch of the field layout is provided in Supplementary Figure 1. We planted sorghum in plots that are naturally infested with *Striga* in two field sites (Kumi and Bukedea) in eastern Uganda and one plot in Western Kenya (Alupe) for two seasons. The experiment was replicated three times in each of the locations using a completely randomized block design (CRBD). For control experiments, sorghum accessions were simultaneously planted in *Striga*-free plots within the same locations.

Each accession was planted in a sub-plot (size 3.2 × 2.5 m) separated by a one-meter footpath at a spacing of 80 cm between rows and 30 cm between plants (Supplementary Figure 1). To ensure uniform *Striga* infection in the infested fields, artificial inoculation with *Striga* seeds harvested from the same and adjacent fields was conducted. At planting, each hill was infested with approximately 3000 *Striga* seeds that were prepared by mixing 5 g of *Striga* seeds with 5 kg of washed sand and 1 tablespoon of inoculum and applied to each hill according to the methods of Jamil et al. (2013). Each accession was planted in five rows and five hills per row at a population density of 80,000 plants ha^−1^. In addition, 50 kg ha^−1^ of diammonium phosphate (DAP) fertilizer were applied. To ensure there were no gaps, three seeds were sown in each hole and thinned after 21 days, which left only one plant per hole. The first weeding was done 21 days after sowing using a hoe while subsequent weeding was conducted by hand pulling to avoid disturbing the emerging *Striga* plants.

### Statistical data analysis

The generalized linear model (GLM) implemented in SAS version 9.1 was used for analysis of *Striga* resistance data from the laboratory experiments. Analysis of variance (ANOVA) was performed to compare the means for biomass, length and number of infecting *Striga* and to fit a factorial ANOVA for each replicate across the night accessions. In addition, Tukey's honest significant difference (HSD) test was performed to calculate mean separations. These data were presented as relative means ± SD in the form of graphs using Graph Pad Prism version 6 (http://www.graphpad.com). In addition, mean *Striga* attachments, length, and biomass data from sorghum accessions against the 3 *Striga* ecotypes were clustered and significant values (*p* ≤ 0.05) were visualized as a heat map using a custom hierarchical clustering R script. Finally, the median length data were used to generate a heat map as described above.

All nine accessions (the same ones used in laboratory screening) were evaluated for the number of *Striga* plants emerging 44, 58, 72, and 86 days after planting. Successive *Striga* counts were used to calculate the “Area under *Striga* number progression curve” (AUSNPC) as described by Rodenburg et al. (2005). The grain yield of sorghum in infected and non-infected fields from three randomly selected plants was weighed, and an average was obtained to provide the yield per plant in the nine sorghum accessions. This was then extrapolated to the yield in kg ha^−1^. The Percentage yield reduction was calculated as a function of the difference between the yield in *Striga*-free and *Striga*-infested fields. In addition, a *t*-test was used to determine the significant yield reduction as a result of *Striga* infestation. For AUSNPC and mean yields, ANOVA followed by Tukey's HSD test was performed to determine the mean separations.

## Results

### Resistance response of wild sorghum accessions under controlled laboratory conditions

**Figure 1:**
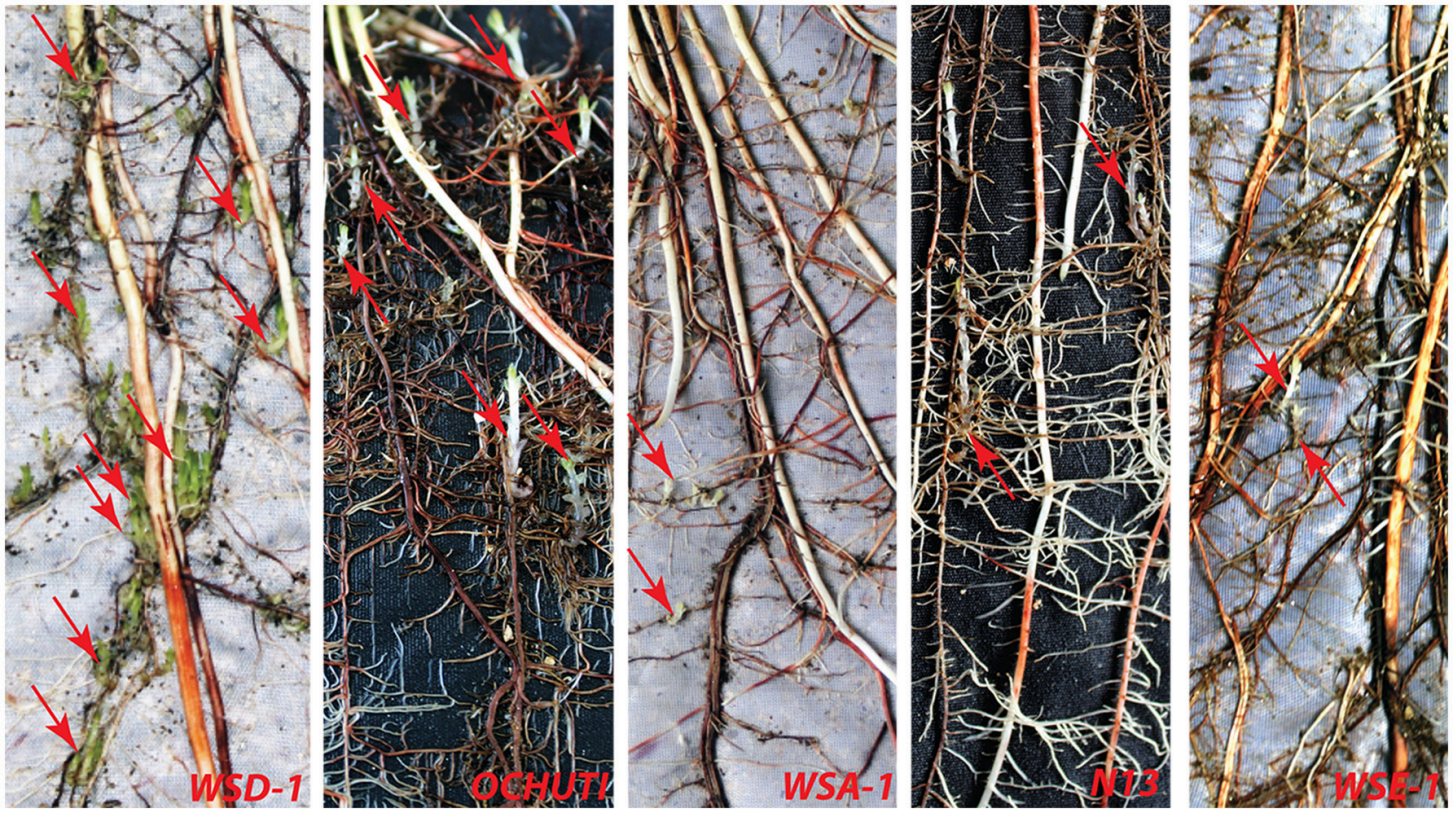
**Profile of differential ***Striga hermonthica*** attachments in wild sorghum accessions: WSD-1, Ochuti, WSA-1, N13, and WSE-1 21 days following infection of host roots with ***Striga*** seeds collected from Kibos**. Red arrows indicate attachment points to the host by the parasite. Susceptible accessions represented here by WSD-1 and Ochuti showed a high number of *Striga* attachments compared to the control N13. Resistant accessions represented here by WSA-1 and WSE-1 showed few *Striga* attachments.

*Striga* infection in various sorghum hosts was variable with respect to the number, length, and biomass of *Striga* attachments. These three metrics were used to determine the resistance of wild sorghum. Susceptible sorghum had numerous *Striga* attachments. A representation of the resistance response of wild sorghum is provided in Figure 1.

**Figure 2:**
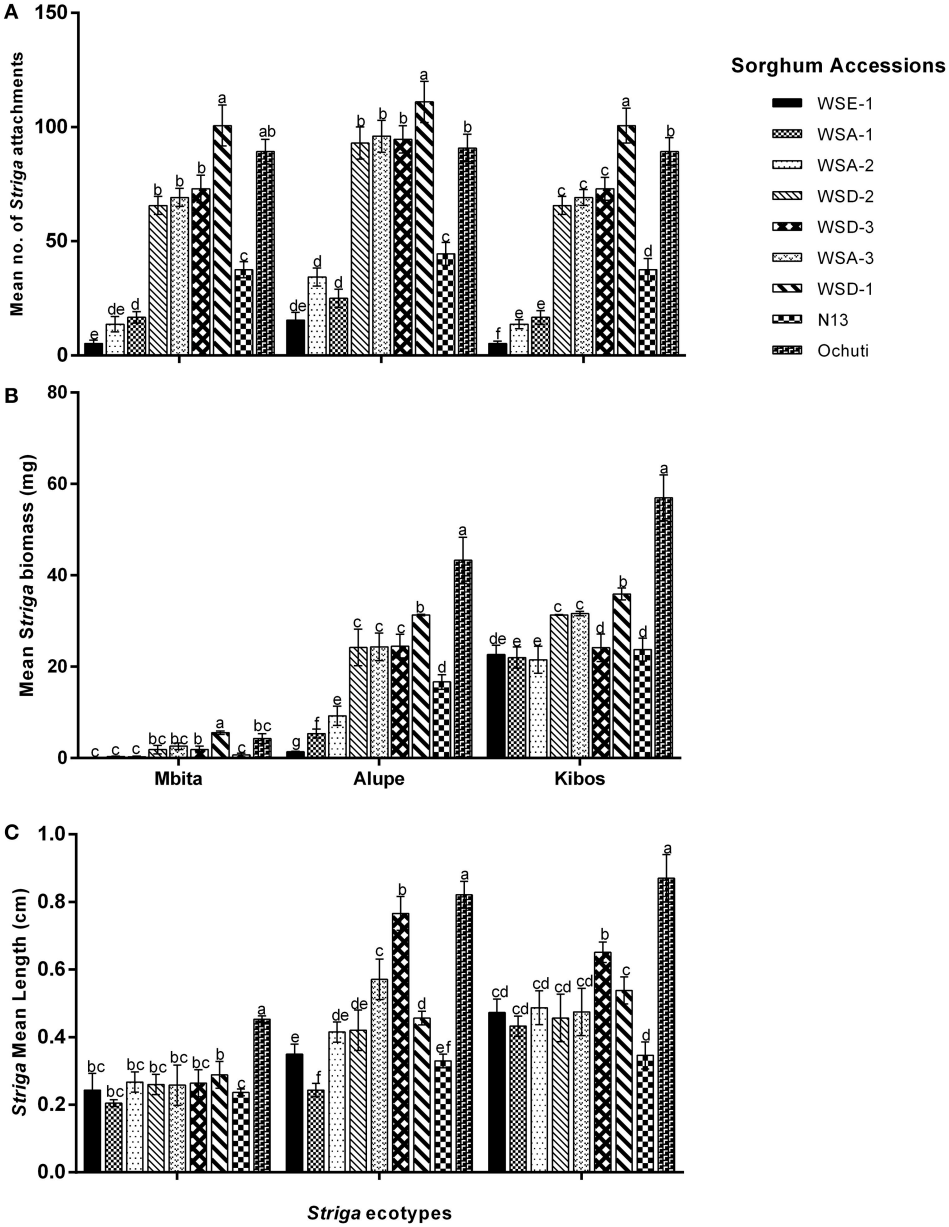
**Resistance response of sorghum accessions to ***Striga hermonthica***: (A)** Mean *Striga* attachments; **(B)** Mean bioass of *Striga* plants; **(C)** mean *Striga* length on wild sorghum accessions using 3 ecotypes of *Striga*. The number of *Striga* plants was measured 21 days after infection of sorghum roots with *S. hermonthica* seeds. Vertical bars represent the mean ±SD while letters represent mean separations at *p* ≤ 0.05. The values given represent the mean for each plant.

We observed a significantly higher level of resistance between three sorghum accessions (WSE-1, WSA-1, and WSA-2) and the positive control N13. These three accessions had the lowest number of *Striga* attachments (Figure 2A) and the lowest *Striga* biomass (Figure 2B). The low number of *Striga* attachments and biomass for WSE-1, WSA-1, and WSA-2 were consistently replicated for each *Striga* ecotype infecting the sorghum accessions (Figures 2A,B). Among the three most resistant accessions, there were significant differences in mean *Striga* attachments between WSE-1 and WSA-1 when only *Striga* from Kibos was used. However, there were significant differences in mean *Striga* biomass among the accessions (WSE-1, WSA-1, and WSA-2) when the sorghum accessions were infected with the Alupe *Striga* ecotype (Figures 2A,B).

**Figure 3:**
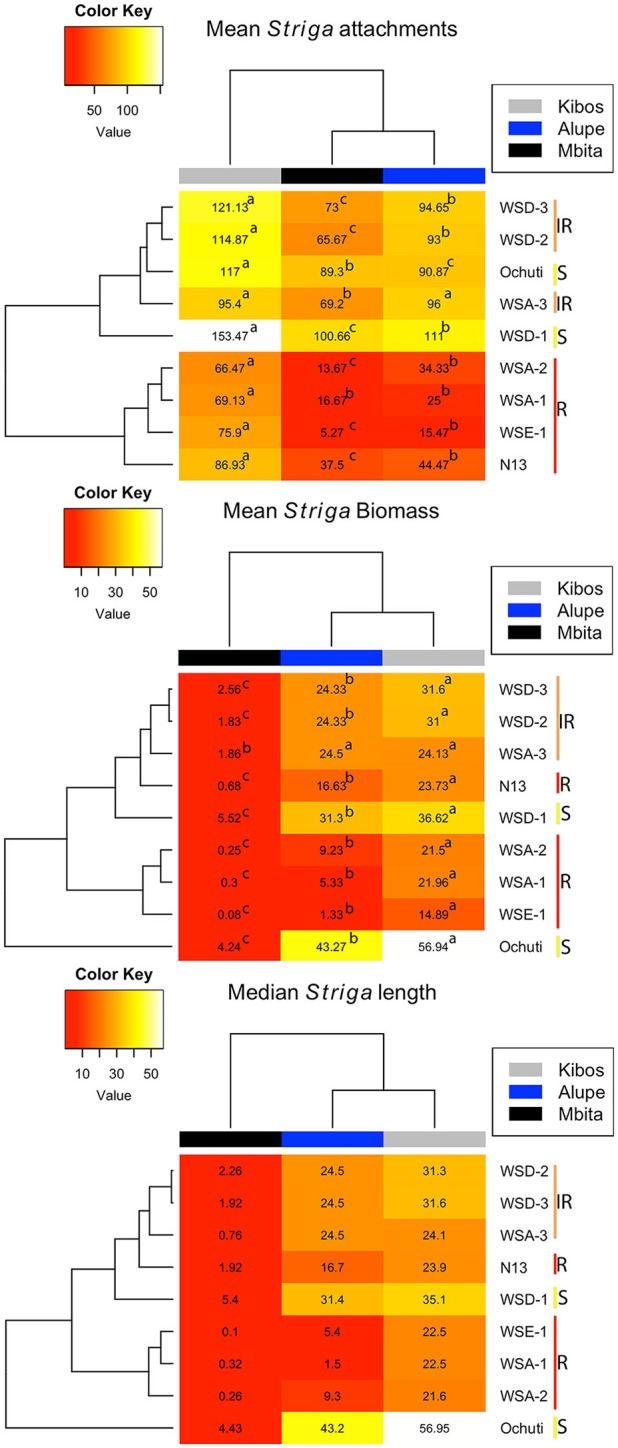
**Heatmaps (generated using mean ***Striga*** attachments, biomass and median ***Striga*** length) showing levels of resistance of sorghum accessions infected with ***Striga*** seedlings**. The yellow bar labeled “S” (Ochuti and WSD-1) represents the most susceptible accessions, and the orange bar labeled “IR” is a group consisting of WSA-3, WSD-2, and WSD-3 that had intermediate resistance, while the red bar labeled “R” represents the most resistant group, which consists of WSA-1, WSA-2 WSE-1, and N13. The heatmaps also provide an indication of the virulence of *Striga* ecotypes. More attachments, more biomass and longer *Striga* seedlings formed from Kibos and Alupe ecotypes compared to Mbita. The letters indicate significance differences between ecotypes on the same accession at *p* < 0.05.

Despite the variations observed with regard to *Striga* attachments and biomass among the resistant and susceptible sorghum, we did not observe those variations with regard to *Striga* length (Figure 2, Supplementary Figure 2). For example, in Mbita, there were only significant differences between the mean *Striga* length of Ochuti, WSD-1 and the rest of the accessions. Similarly, for Kibos, there were significant differences in *Striga* length between Ochuti—which had the highest length—and the accession with the next highest length, WSA-3. WSA-3 had a significantly higher *Striga* length than the rest of accessions. However, there were more significant variations with regard to length when *Striga* from Alupe was used. In this case we found that: (i) WSE-1 and WSA-1 had the shortest *Striga* seedlings attached to them; (ii) *Striga* seedlings attached to WSA-1 were significantly shorter than those attached to WSE-1 and; (iii) the length of *Striga* seedlings attached on N13 were statistically similar to those on WSA-1 and WSE-1. For Alupe, Ochuti had the highest mean length, which was significantly different compared to the accession that ranked second in *Striga* mean length, WSD-1. Remarkably, when we used median *Striga* length to rank and cluster resistance in sorghum, we obtained a heatmap that was strikingly similar to the one generated using biomass (Figure 3).

We determined *Striga* resistance rankings based on mean *Striga* count and biomass because of the consistency of these metrics across the sorghum accessions and *Striga* ecotypes. In general, three groups of resistance with respect to N13 emerged: a highly resistant group comprised of WSE-1, WSA-1, and WSA-2; an intermediate resistance group comprised of WSA-3, WSD-2, and WSD-3, and a highly susceptible group with WSD-1 and Ochuti (Figures 2, 3).

Regarding *Striga* virulence, we found that the three *Striga* ecotypes—Kibos, Alupe, and Mbita—exhibited significant variations. There were significantly higher numbers of *Striga* attachments for all sorghum accessions except WSA-3 when they were infected with the Kibos ecotype. When we compared Mbita and Alupe ecotypes on how they infected sorghum, we found that the Alupe ecotype induced significantly more attachments in WSD-3, WSA-3, WSA-2, WSE-1, and N13 but not in Ochuti and WSD-2. For *Striga* biomass, all sorghum accessions had significantly more biomass when they were infected with all *Striga* ecotypes in the order of Kibos, Alupe, and Mbita. Similarly, the lengths of *Striga* seedlings attached to sorghum were ranked by virulence in the order of Kibos, Alupe, and Mbita. Six sorghum accessions (WSA-2, WSE-1, WSA-1, WSD-3, and WSD-1) showed significantly longer *Striga* when they were infected with Kibos seeds compared to Alupe. When the virulence of Alupe and Mbita were compared in sorghum with regard to length, all accessions had significantly longer *Striga* with regard to Alupe *Striga*.

Taken together, our results suggested that for the accessions that were screened, aethiopicum and the arundenaceum races of wild sorghum exhibited higher levels of resistance compared to the drummondii races. In particular, WSE-1, WSA-1, and WSA-2 could provide valuable resistance against *S. hermonthica*. Additionally, the resistance of these accessions was effective against three of the most common *Striga* ecotypes in Kenya, which indicated that the resistance had a broad spectrum.

### Wild sorghum accessions blocked *Striga* penetration through mechanical and possible biochemical barriers

**Figure 4:**
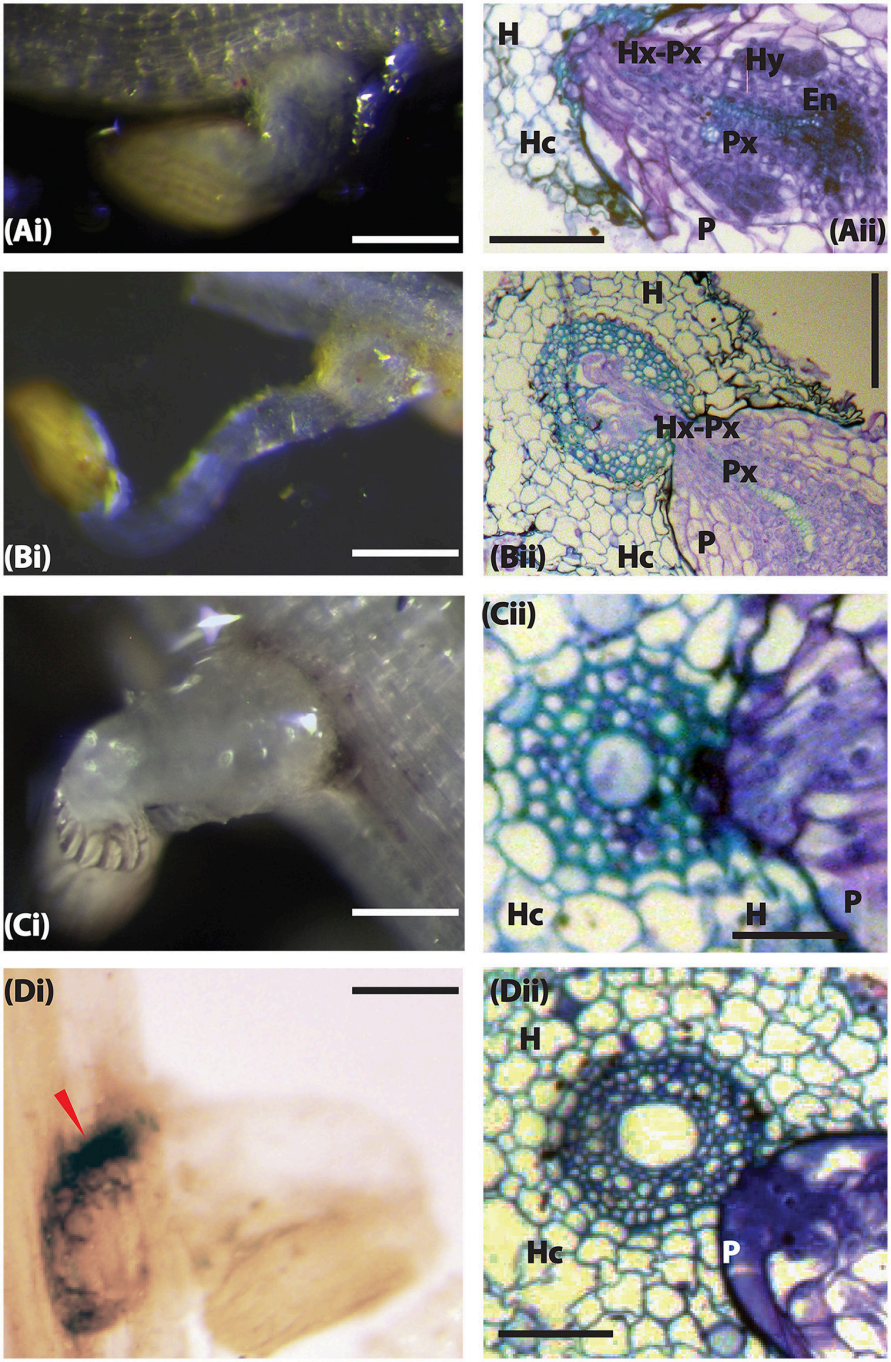
**The host resistance mechanism of sorghum to ***Striga hermonthica*** 3 DAI. (A**_i_**)** Colonization of WSD-1 sorghum root by *S. hermonthica* (Kibos ecotype) showing a well-established haustorium (scale bar 1 mm). **(A**_ii_**)** Transverse section of an embedded root tissue of WSD-1 sorghum accession 3 days after infection with showing penetration of the host root cortex and endodermis as well as connections between the host and parasite xylem (Hx-Px). The scale bar is 0.1 mm. **(B**_i_**)** Genotype N13. The haustorium is well-developed and shows swelling at the point of attachment. The scale bar is 1 mm. **(B**_ii_**)** Transverse section of embedded tissue from N13. By this time, the parasite had started developing vascular connections. The scale bar is 0.1 mm. **(C**_i_**)** A resistant wild sorghum accession (WSA-1). The scale bar is 5 mm. **(C**_ii_**)** A transverse section of the resistant wild sorghum accession WSA-1. *Striga* was not able to penetrate the host endodermis to make vascular connections. The scale bar is 0.1 mm. **(D**_i_**)** Colonization of WSE-1 had more intense phenolic deposits (red arrow). The scale bar is 5 mm. **(D**_ii_**)** A transverse section through the haustorium of the resistant wild sorghum accession WSE-1.

*Striga* infects its host by using a haustorium that connects the parasite with the host through xylem vessels (Dörr, 1997). To understand how wild sorghum accessions resisted *Striga* parasitism, we did microscopic observations of *Striga* haustorium at 3 and 9 DAI. At 3 DAI, *Striga* had successfully penetrated all accessions except for WSA-1, WSA-2, and WSE-1. In Figures 3, 4, we showed the resistance of WSA-1 and WSE-1 compared to the resistant control N13. We also showed a susceptible interaction represented by WSD-1.

**Figure 5:**
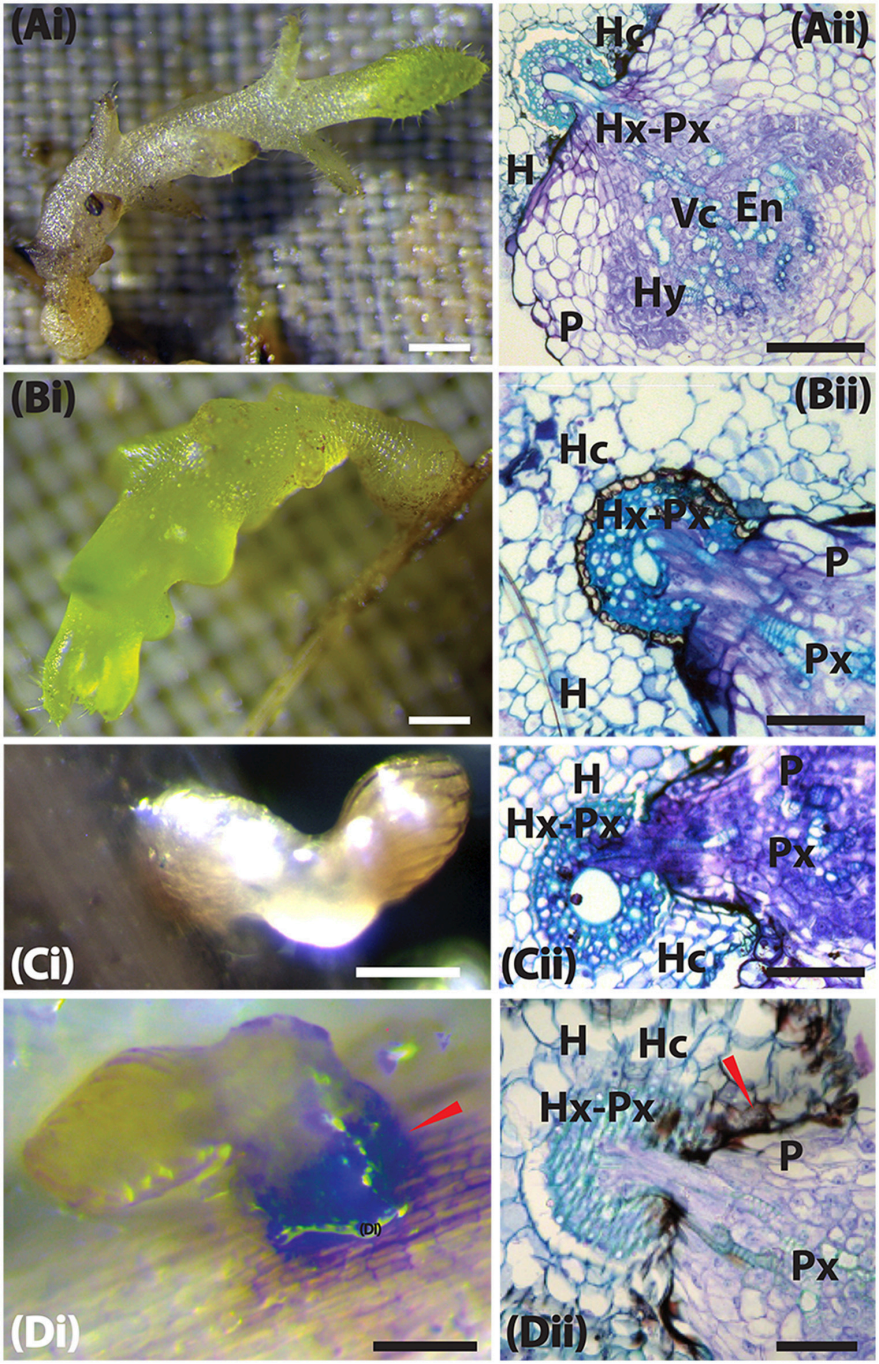
**Host resistance mechanism of sorghum to ***Striga hermonthica*** 9 DAI. (A_**i**_)** Colonization of WSD-1 sorghum root by *S. hermonthica* (Kibos ecotype) 3 days after infection showing a well-established haustorium (the scale bar is 1 mm). **(A**_ii_**)** Transverse section of an embedded root tissue of WSD-1 sorghum accession showing penetration of the host root cortex and endodermis and vascular connections between the host and parasite xylem (Hx-Px). Scale bar is 0.1 mm. **(B**_i_**)** Genotype N13 9 days after infection. The haustorium is well-developed and shows swelling at the point of infection. The scale bar is 1 mm. **(B**_ii_**)** Transverse section of embedded tissue of N13 9 DAI. By this time, the parasite had a well-developed endophyte (En) and hyaline body (Hy). The dark areas around the pericycle may indicate a resistance mechanism. The scale bar is 0.1 mm. **(C**_i_**)** A resistant wild sorghum accession (WSA-1) infected with *S. hermonthica*. The parasite was weak and did not form much vegetative tissue. The scale bar is 5 mm. **(C**_ii_**)** A transverse section of a resistant wild sorghum accession (WSA-1). Only a small part of the parasite was able to penetrate the host vascular system. The scale bar is 0.1 mm. **(D**_i_**)** Colonization of WSE-1 9 DAI showing more intensive secondary metabolite deposits (red arrow). In most cases, the parasite died and did not persist past 14 DAI. The scale bar is 5 mm. **(D**_ii_**)** A transverse section through the haustorium of the resistant wild sorghum accession WSE-1. Only a small part of the parasite xylem was able to make vascular connections with the host. In addition, the hyaline body (Hy) as well as the endophyte (En) were much smaller compared to N13. The scale bar is 0.1 mm.

In susceptible interactions, attachment, and subsequent formation of vascular connections was characterized by the swelling of the *Striga* radicle at the point of contact with the host roots (Figure 4A_i_). Histological analysis of this section revealed a swollen haustorium with a well-differentiated *Striga* xylem that had already connected with the sorghum xylem (Figure 4A_ii_). As the infection progressed (at 9 DAI), the parasite's vegetative tissue grew vigorously, and the haustorium significantly expanded for interactions with susceptible sorghum and N13 (Figures 5A_i_, B_i_). A transverse section through a haustorium at 9 DAI showed that it was well-developed with a hyaline body (Hy), a vascular core (Vc) consisting of the xylem vessels, and an endophyte (En) that entered the host root cortex and endodermis (Figures 5A_ii_, B_ii_). This progression of infection and haustorium development was typical of most accessions. For the resistant control N13, we observed extra thickening on the pericycle (Figures 4B_ii_, 5B_ii_).

Among the three resistant wild sorghum accessions (WSE-1, WSA-1, and WSA-2), the haustorium did not make vascular connections by 3 DAI. These connections only occurred for a few attachments. The haustorium of the parasite invading a resistant accession did not display any swelling, and as histological analyses revealed, the haustorium did not penetrate the host endodermis to make vascular connections with the host (Figures 4C_ii_, D_ii_, 5C_ii_, D_ii_). This lack of connections was the case for WSA-1, WSA-2, and WSE-1. In WSA-1 and WSA-2, only a small section of the parasite haustorium had penetrated the host endodermis and made vascular connections (Figure 5C_ii_). We also observed deep blue staining at the point of contact between the *Striga* haustorium and the host endodermis for both WSA-1 and WSA-2 (Figures 4C_ii_, 5C_ii_). Additionally, WSE-1 showed deposits of secondary metabolites that caused an intense colouration at the site of parasite attachment (Figure 4D_i_) as early as 3 DAI. Most *Striga* attached to WSE-1 died within the first few days of attachment. Those attachments that persisted at 9 DAI showed intense coloration and a poorly developed *Striga* plant (Figures 5C_i_,D_i_). A transverse section through a haustorium from this time point showed deposits of secondary metabolites characterized by increased colouration and limited vascular connections (Figure 5D_ii_).

These results suggest that the mechanical barriers that inhibited ingression of the haustorium into host tissue may be the cause of resistance in wild sorghum. The results from this experiment also suggested that the interactions between WSE-1 and *S. hermonthica* could be mediated by host-parasite biochemical integrations that lead to deposits of secondary metabolites.

### Resistance response of wild sorghum accessions under natural *Striga* infestation

**Figure 6:**
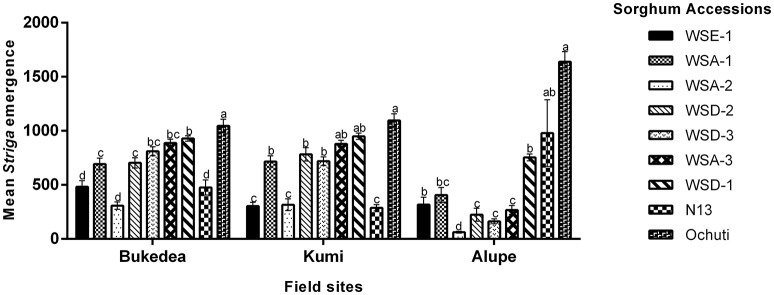
**The resistance response of sorghum accessions to ***Striga hermonthica*** at the Kumi, Bukedea, and Alupe field sites**. Mean *Striga* emergence was determined using AUSNPC. The vertical bars represent the mean ± SD while the letters (above each bar) indicate the mean separation and field sites, respectively, at *p* ≤ 0.05.

Under natural *Striga* infestation, we made the following general observations: (i) The three field sites (Kumi, Bukedea, and Alupe) had significant differences in their AUSNPC, which alluded to differences in *Striga* virulence at these sites; (ii) WSE-1 and WSA-2 were the most resistant sorghum accessions in both Kumi and Bukedea but not in Alupe and; (iii) Ochuti and WSD-1 were the least resistant accessions for all of the field sites. These results are presented in Figure 6.

In Kumi, WSE-1 and WSA-2 had similar resistance to N13 (Figure 6). Similarly, in Bukedea, the most resistant accessions were WSA-2, WSE-1, and N13. The next four accessions: WSD-2, WSD-3, WSA-1, WSA-3, and Ochuti had similar resistance responses in Kumi. The same accessions (WSD-2. WSD-3, WSA-1, and WSA-3) had similar resistance in Bukedea. At this site, Ochuti was the most susceptible sorghum (Figure 6).

In Alupe—similar to Kumi and Bukedea—the most resistant accession was WSA-2. However, with an AUSPNC of 64.17 ± 17.83, this accession showed a significantly higher resistance compared to WSD-2 (AUSPNC, 182 ± 110.05) and WSD-3 (AUSPNC, 166.33 ± 53.79). The next cluster of accessions consisted of N13, WSE-1, WSA-1, and WSA-3 (Figure 6, Supplementary Table 1). In both Kumi and Bukedea, the least resistant accession was WSD-1 and the susceptible check Ochuti, although in Alupe N13 recorded a similar profile of emergence (Figure 6, Supplementary Table 1). Similar to Kumi and Bukedea, the most resistant sorghum accession was WSA-2. This resistance was significantly different from WSD-2, WSD-3, and WSA-3, which were the accessions ranked second, third, and fourth in terms of resistance at this field site. N13 had a significantly lower resistance than this group of accessions (Figure 6).

In summary, the field experiments confirmed the laboratory results that wild sorghum harbored resistance to *Striga*. In particular, aethiopicum (WSE-1) and arundinaceum (WSA-2) showed resistance comparable to the resistance of the control, N13, at all field sites except Alupe. In addition, the three field sites showed consistency in the resistance response of sorghum accessions to *Striga*.

### Effects of *Striga* infestation on sorghum yield

**Figure 7:**
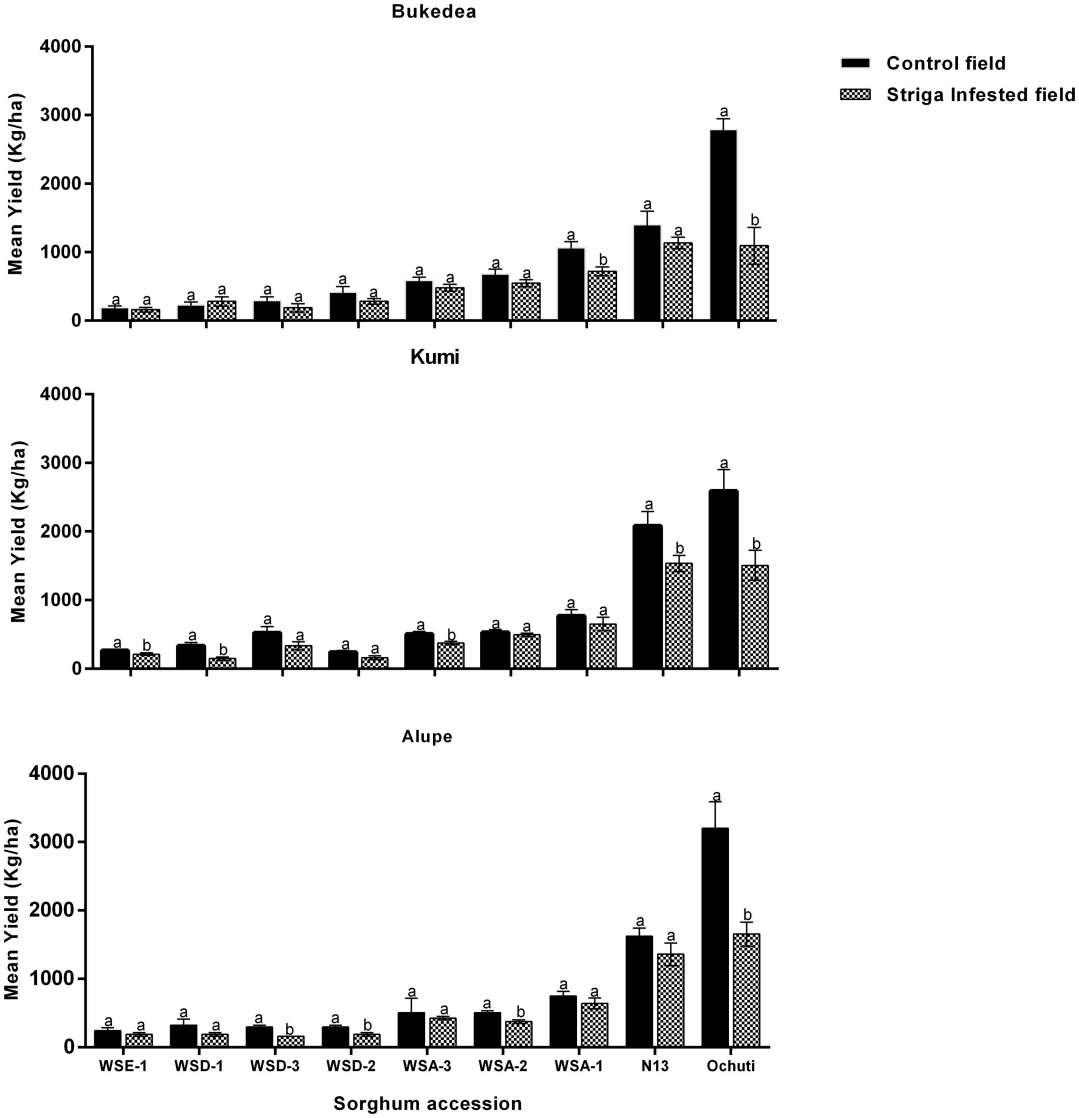
**Yield performance of sorghum accessions at field sites under ***Striga*** and ***Striga***-free (control) conditions**. The vertical bars represent the mean ± SD (*p* ≤ 0.05) and different letters show presence of significant variations between the control and the *Striga*-infested fields.

**Figure 8:**
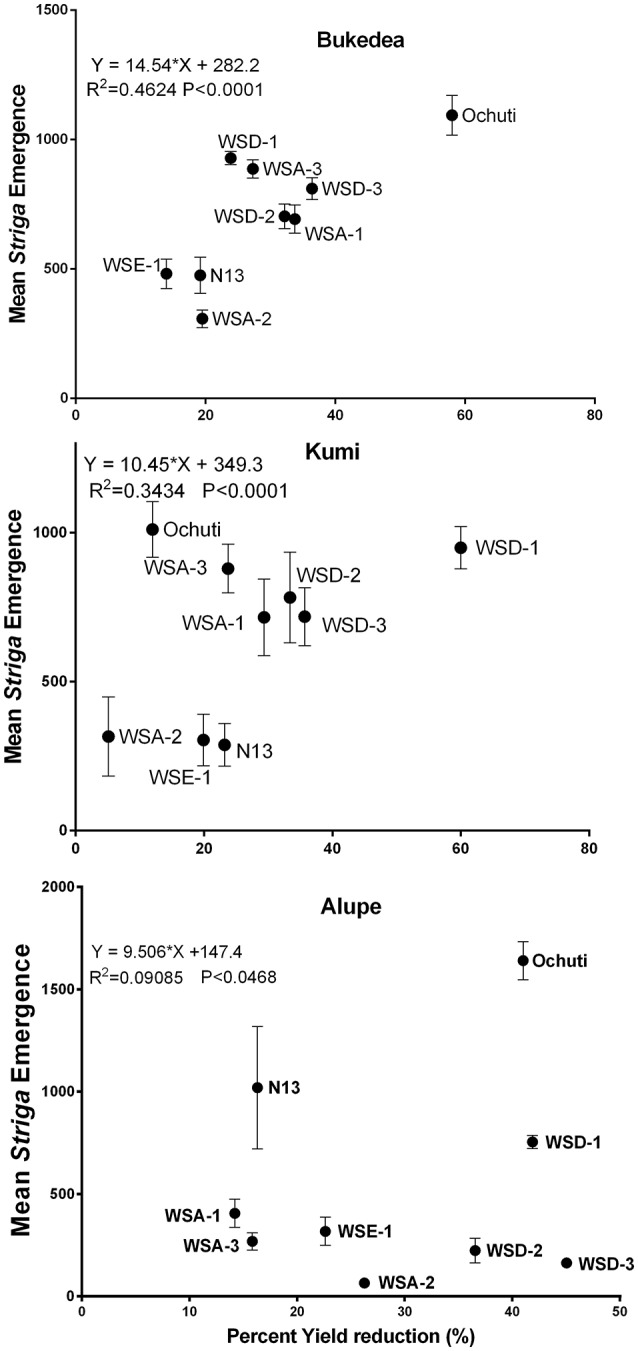
**Correlation of ***Striga*** emergence to percentage yield loss at the three sites**. The vertical axis represents mean emergence (AUSPNC) at 86 days after planting while the horizontal axis represents the percentage yield loss expressed as a yield loss between *Striga*-free fields and yield in *Striga*-infested fields as a function of yield in a *Striga*-free field. The data are presented as the means ± SD.

We assessed the yield performance of sorghum accessions for the reduction of yield due to *Striga* infestation and found the following: (i) Wild sorghum accessions yielded less compared to N13 in all field sites and (ii) *Striga* negatively affected the yields of all sorghum but more severely affected the susceptible accessions. These results are described in Figures 7, 8.

Yields of wild sorghum accessions in *Striga*-free plots varied significantly among one another with the average of the lowest yielding accessions being WSE-1 (230.9 kgha^−1^) compared to the highest Ochuti with an average yield of 2857.8 kgha^−1^ (Figure 7). Despite these variations, the yield of a particular accession did not vary with site. In other words, the yields of each accession were similar for all three field sites without statistical significance.

*Striga* had a negative effect on yield for all accessions. For some accessions, the effects of *Striga* infestation were more significant and depended on the field trial site. For example, *Striga* infestation only reduced the yields for N13 in Kumi (23.21 ± 2.00%) with statistical significance but not in Alupe or Bukedea (Figures 7, 8). For the most resistant wild accession, WSE-1, the percentage yield reduction was statistically significant only at the Kumi field site. Conversely, the yield reductions were more severe in the susceptible accessions (Figure 7). When considering the susceptible control (Ochuti), the percentage yield reduction was most dramatic in Alupe (48.33%), followed by Bukedea (41.75%) and Kumi (24.88%). These results suggest that *Striga* altered the yields of susceptible accessions more drastically compared to resistant accessions.

We further described the relationship between the percentage yield reduction and *Striga* infestation using regression analysis, and the results are shown in Figure 8. We observed a general positive relationship between *Striga* emergence and percentage yield reduction as well as a negative correlation between host resistance and percentage yield reduction.

The correlation between *Striga* emergence and yield reduction was more significant in Bukedea (*R*^2^ = *0.4624; p* < *0.0001*) and Kumi (*R*^2^ = *0.2136; p* < *0.0001*) compared to Alupe (*R*^2^ = *0.007; p* = *0.047*). In general, resistant accessions (WSE-1, WSA-2) had low *Striga* emergence and the lowest yield reduction, while for the susceptible accessions, including Ochuti, WSD-1, and WSD-3, the yield reduction became more severe with *Striga* emergence.

## Discussion

Our goal was to determine to what extent wild sorghum could provide resistance to the parasitic weed *Striga* compared to the resistant cultivar N13. Successful parasitism by *Striga* in host roots was manifested by multiple, fast growing *Striga* attachments, i.e., high virulence. Accordingly, a few small *Striga* plants parasitizing host roots indicated a resistant host that did not support the parasite. We showed that: (i) Under laboratory conditions, wild sorghum accessions WSE-1, WSA-1, and WSA-2 have significantly higher resistance than the current *Striga* resistant control line—N13; (ii) Under field conditions, WSE-1 and WSA-2 have resistance comparable to that of N13 and; (iii) the resistance in wild sorghum occurred because *Striga* was unable to penetrate the host's endodermis and make vascular connections due to mechanical and/or biochemical barriers.

Pertaining to analyses of *Striga* resistance in the laboratory, we showed that wild sorghum accessions WSE-1, WSA-1, and WSA-2 were highly resistant against *S. hermonthica* compared to N13. The rankings for wild sorghum resistance had an overall similar pattern for all three post-attachment metrics of *Striga* resistance: *Striga* length, number and biomass. Previous studies using rhizotrons to determine *Striga* resistance in hosts have used these metrics with results comparable to ours (Gurney et al., 2006; Cissoko et al., 2011). For example Pescott (2013) found a mean of 75 attachments on the resistant sorghum genotype Brhan compared to our mean of 56 on N13. In rice, the most resistant rice cultivar (Nipponbare) averaged 30 attachments in a study conducted by Cissoko et al. (2011).

One result that was noteworthy was that although the mean number of attachments and mean biomass gave the same level/pattern of resistance and virulence, it was not possible to determine the resistance of sorghum using the mean length of *Striga* attachments. This outcome likely occurred because of the variability in *Striga* sizes. For example, a sorghum accession could have multiple small-sized attachments and one or two long attachments. This variation likely skewed the data and gave the impression that the accession was resistant. For this reason, mean *Striga* biomass seemed to be a good measure of resistance because it took both the number of attached parasites as well as their size into account. Interestingly, when we used median length to rank the resistance of sorghum accessions and presented the results in a heat map, we got the same pattern as we got for biomass. This result suggested that median length rather than mean length could be a better method to rank resistance if the *Striga* seedling length was highly variable.

With regard to mechanism of resistance in the wild sorghum accessions, we found that *Striga* had delayed penetration into the host endodermis for WSE-1, WSA-1, and WSA-2. In addition, we found that for WSE-1 Striga parasitism induced secretion of large amounts of secondary metabolites, which were probably phenolic compounds. These resistance mechanisms have important implications as platforms for further genetic improvement of cultivated sorghum for *Striga* resistance.

We described the resistance mechanisms of WSE-1, WSA-1, and WSA-2 as qualitative, and the mechanisms were similar to N13. Like N13, these accessions slowed the ability of *Striga* to penetrate host tissue and make vascular connections. For *Striga*, successful parasitism must involve overcoming mechanical barriers, such as the host's cell wall. This resistance—which varied from host to host—could be a manifestation of physiological and/or the biochemical incompatibility of the parasite growth on its host (Yoshida and Shirasu, 2009). The biological and genetic mechanisms underpinning this form of resistance in *Striga* are not completely understood but it is plausible that the effect was the result of multiple genes acting to fortify the host against invasion by the parasite. Such mechanisms include thickening of cell walls in the pericycle, lignification and silica deposition, which was suggested in Maiti et al. (1984). This form of resistance, which is exemplified by N13, has led to identification of several Quantitative Trail Loci (QTL) by mapping populations of resistant N13 and susceptible E103 (Haussmann et al., 2004). These QTL have been integrated into breeding programmes in Africa (Masiga et al., 2014; Mohamed et al., 2014; Yohannes et al., 2015). Therefore, for quantitative *Striga* resistance comparable to N13, WSE-1, WSA-1, and WSA-2 are good candidate accessions that could be integrated into varieties preferred by farmers. Additionally, these accessions could be used in the identification of *Striga* resistance loci by exploiting new and high throughput genotyping technologies, such as high-throughput sequencing. Therefore, unique markers could be identified, which would facilitate marker-assisted breeding of *Striga* resistance in sorghum.

Additionally, we singled out the resistance shown by WSE-1 as being mediated by biochemical reactions elicited by the parasite. Although this mechanism is not well-characterized in parasitic plants, this defense mechanism is reminiscent of studies that have shown chemical molecules could be produced as needed during pathogen attack (inducible) or starting with a small amount of preformed metabolites (Lanoue et al., 2010; Wurst et al., 2010). Some studies have further demonstrated the role of root secondary metabolites in induced plant defenses. For example, *Ocimum basilicum* secretes rosmarinic acid upon attack by the pathogenic fungus *Pythium ultimum* (Bais et al., 2002) and induction of iridoid glycosides in root exudates of *Plantago lanceolata* in the presence of nematodes was reported in Wurst et al. (2010). The induction of biochemical compounds—which was characterized by deep colorations at the host-parasite interphase in the resistant sorghum accession WSE-1 could be suggestive of a resistance mediated by these molecules. Further biochemical studies—such as metabolomics in a recent review (van Dam and Bouwmeester, 2016)—will be required to further identify the role of metabolites in *Striga*-host interactions.

Laboratory analysis provided the unique opportunity to minimize environmental variations and ensure that the resistance rankings that were observed could be confidently associated with the genotype. However, these data did not always translate to the field because other factors may influence resistance through genetic χ environment interactions, which have been observed before for *Striga*. These interactions were demonstrated in a study (Cissoko et al., 2011), who found an upland rice variety NERICA4 that was resistant using rhizotron assays. NERICA4 was, however, found to be susceptible under field conditions (Atera et al., 2012).

We therefore complemented our laboratory observations with field trial data. From the wide array of tools that are traditionally used to measure *Striga* virulence and the host resistance (Omanya et al., 2004; Rodenburg et al., 2005) at the field level, we determined AUSPNC, which was derived by integrating the curve of *Striga* emergence over time.

Strikingly, the resistance rankings of sorghum accessions screened in the laboratory were similar to those found with field experiments—for all field sites—with WSE-1, WSA-2 showing the most resistance. Additionally, the two least resistant accessions in all field sites were consistently WSD-1 and Ochuti. However, in Alupe N13 did not display the level of resistance observed in Kumi and Bukedea. In fact, the AUSPNC of N13 in Alupe was similar to that of Ochuti. Previous studies involving N13 in Kumi and Bukedea obtained an AUSPNC that averaged <500 (Olupot, 2011) which, is comparable to what we obtained—476 ± 70 and 288.17 ± 29.11 in Bukedea and Kumi respectively. This was lower than the AUSPNC of N13 in Alupe (978.83 ± 308.36). The variability in response to *Striga* infestation by site in our field experiments could be attributed to environmental effects such as different climatic conditions, differences in soil types or factors inherent to *Striga* characteristics such as seedbank or virulence.

With regard to the variability in *Striga* virulence, our rhizotron assays showed that the *Striga* ecotype from Alupe was significantly more virulent compared to Mbita but was less virulent than the ecotype from Kibos. This result showed there were variable differences in the virulence of Alupe seed ecotypes. In other words, even within the same *Striga* ecotype, variations in virulence occurred. This apparent lack of a clear association between virulence and *Striga* ecotypes supports the heterogeneity of *S. hermonthica* observed by Pescott (2013). Unlike *S. asiatica*, which has a highly inbreeding mating system (Gethi et al., 2005), and *S. gesneroides*, which has distinct race structures (Li et al., 2009), *S. hermonthica* has high outcrossing (Gethi et al., 2005), and that makes virulence grouping among ecotypes very nebulous. Therefore, it has been difficult to connect virulence to a specific eco-geographic region.

Another important consideration when comparing laboratory and field experiments for *Striga* resistance is the production of the germination stimulant strigolactone. Laboratory screens use artificially pre-germinated *Striga* seedlings and these screens forfeit the opportunity to test for pre-*Striga* germination resistance as well as resistance during other belowground stages (e.g., germination, attachment, below-ground development). In contrast, in field infestations, the host must produce enough strigolactone to allow *Striga* to germinate. Therefore, field screenings test for both pre- and post-*Striga* germination resistance. As such, it is important to establish the strigolactone profile of wild sorghum accessions to determine if they also exhibit low germination stimulant production as a resistance mechanism.

There is another important aspect of the *Striga*-host relationship, which is tolerance, i.e., the ability of a host to sustain a certain yield and experience less damage under *Striga* infestation (Parker and Riches, 1993; Rodenburg et al., 2006). Whereas, a resistant host “fights” the pathogen, a tolerant host “learns to live” with the pathogen thereby ameliorating the damage inflicted by the pathogen. As expected, *Striga* reduced the yields of all sorghum accessions, albeit the reductions in some accessions were not significant. N13, WSE-1, and WSA-1 tolerated *Striga* to the greatest extent in some field sites that did not have any significant yield reductions. In this study, the resistant accessions were also tolerant, which made it difficult to dissociate resistance and tolerance. Rodenburg et al. (2006) observed that tolerance was a complex trait that needs to take into account the resistance of the host as well as the biomass of the infecting *Striga* plants. The authors recommended that tolerance in resistant genotypes be quantified as a reduced yield loss per aboveground *Striga* plant and that the maximum relative yield loss could be used for susceptible genotypes. Further studies will be required to determine the correlation between resistance and tolerance in WSA-1 and WSE-1.

To summarize, our work revealed high *Striga* resistance in wild sorghum accessions. These accessions also had at least two *Striga* resistance mechanisms and therefore underscored the importance of wild sorghum as sources of resistance to *Striga*. Candidate wild sorghum accessions for *Striga* resistance have been identified and are available for multiplication and subsequent improvement. Our work thus sets a technology platform for future genetic improvement of cultivated sorghum. With modern techniques of genetics and genomics, it will soon be possible to “pinpoint” the genetic components that are responsible for resistance in wild sorghum. These insights will facilitate the stacking of appropriate resistance genes/loci in varieties preferred by farmers and *Striga* tolerant cultivars to enhance the durability and stability of defenses over the long-term.

## Author contributions

SR and MT conceived and designed the laboratory experiments. DM, EK, and JM performed laboratory experiments guided by SR and MW. Field experiments were designed and conceived by DO, AM, and NH, the performed by CM. SR, DM, CM, EK, and JM wrote the manuscript. All authors read and approved the final manuscript.

## Funding

The National Academies of Science (NAS) supported this research project under the Partnerships Enhanced Engagement in Research (PEER) program (contract number NAS Sub-Grant Award Letter Agreement Number PGA-2000003439). The Sub-Grant Agreement was funded under Prime Agreement Number AID-OAA-A-11-00012, which was entered into by and between the NAS and the United States Agency for International Development (USAID). MPT was supported by grants from the National Science Foundation (DBI-0701748 and IBN-0322420). We further acknowledge the fieldwork support we received from the International Crops Research Institute for the Semi-Arid Tropics (ICRISAT).

### Conflict of interest statement

The authors declare that the research was conducted in the absence of any commercial or financial relationships that could be construed as a potential conflict of interest.

## Supplementary material

Supplementary Figure 1 **Experimental layout of the field sites**. Each plot was 2.5 m by 3.2 m. Each plot was separated by a 1 m path and the spacing was 80 cm between rows and 30 cm between hills. The whole sorghum field was surrounded by susceptible sorghum (KARI Mtwapa).

Supplementary Figure 2 **A heat map drawn using the *Striga* mean length showing levels of resistance of sorghum accessions infected with *Striga* seedlings**. The yellow bar labeled “S” (Ochuti and WSD-1) represents the most susceptible accessions, the orange bar labeled “IR” is a group consisting of WSA-3, WSD-2 and WSD-3 that had intermediate resistance, while the red bar labeled “R” represents the most resistant group, which consists of WSA-1, WSA-2, WSE-1, and N13. The heat maps also provide an indication of the virulence of *Striga* ecotypes. Longer attachments formed from Kibos and Alupe ecotypes compared to Mbita ecotypes. The letters indicate significances differences between ecotypes for the same accession at *p* < 0.05.

Supplementary Table 1 ***Striga*** **emergence values (Area under *Striga* Number Progressive Curve) of wild and cultivated sorghum accessions across three field sites**.
