# Supplementary figures and images for: Novel Sources of Witchweed (Striga) Resistance from Wild Sorghum Accessions
Source: Front Plant Sci. 2017 Feb 6;8:116. doi: 10.3389/fpls.2017.00116 (PMC5292437; doi:10.3389/fpls.2017.00116)

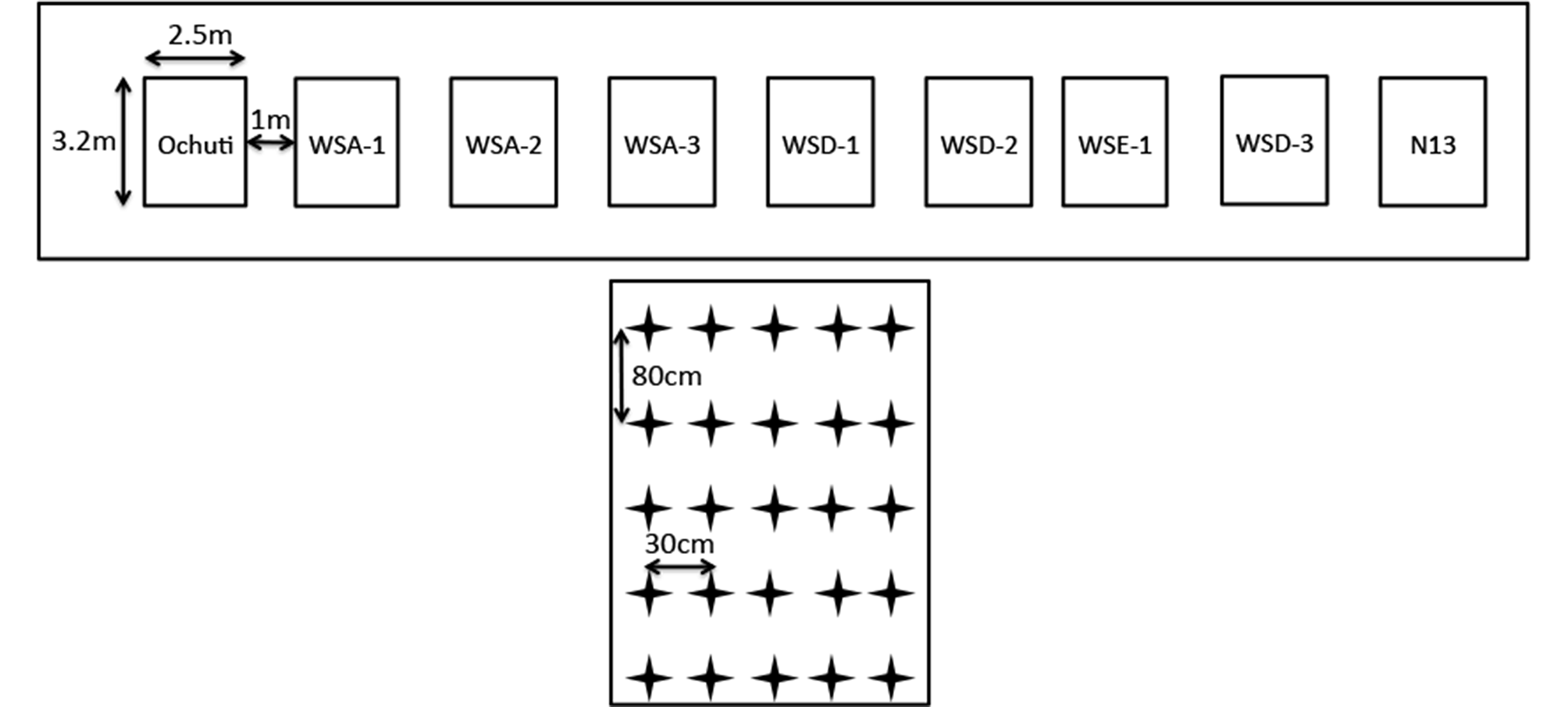

Supplement: Supplementary Figure 1 — Experimental layout of the field sites. Each plot was 2.5 m by 3.2 m. Each plot was separated by a 1 m path and the spacing was 80 cm between rows and 30 cm between hills. The whole sorghum field was surrounded by susceptible sorghum (KARI Mtwapa). [file Image1.TIFF]

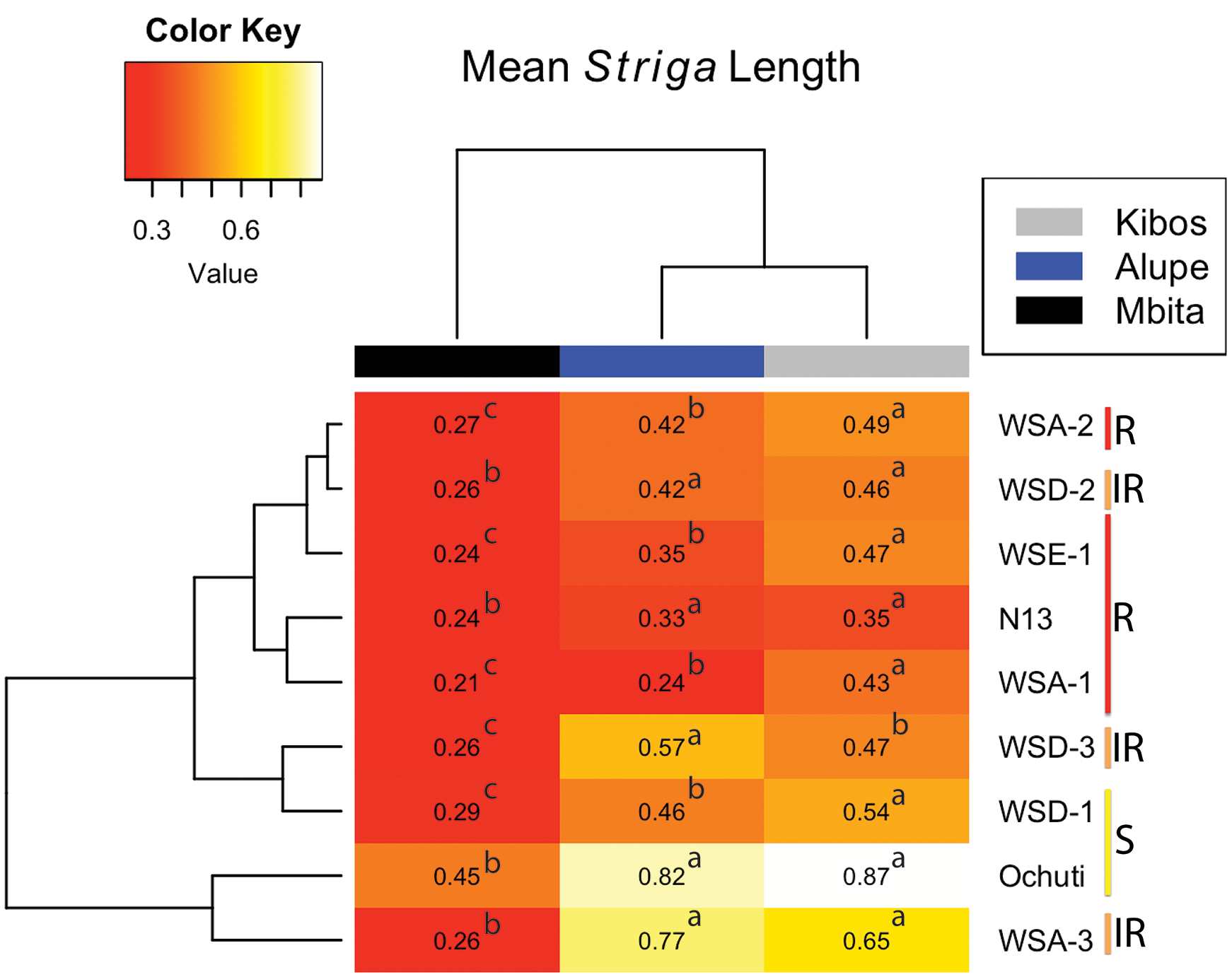

Supplement: Supplementary Figure 2 — A heat map drawn using the Striga mean length showing levels of resistance of sorghum accessions infected with Striga seedlings. The yellow bar labeled “S” (Ochuti and WSD-1) represents the most susceptible accessions, the orange bar labeled “IR” is a group consisting of WSA-3, WSD-2 and WSD-3 that had intermediate resistance, while the red bar labeled “R” represents the most resistant group, which consists of WSA-1, WSA-2, WSE-1, and N13. The heat maps also provide an indication of the virulence of Striga ecotypes. Longer attachments formed from Kibos and Alupe ecotypes compared to Mbita ecotypes. The letters indicate significances differences between ecotypes for the same accession at p < 0.05. [file Image2.TIF]
