# Supplementary material for: Novel Sources of Witchweed (Striga) Resistance from Wild Sorghum Accessions
Source: Front Plant Sci. 2017 Feb 6;8:116. doi: 10.3389/fpls.2017.00116 (PMC5292437; doi:10.3389/fpls.2017.00116)
Supplement: Supplementary Table 1 — Striga emergence values (Area under Striga Number Progressive Curve) of wild and cultivated sorghum accessions across three field sites. [file Table1.docx]

**Supplementary Table 1:** *Striga* emergence values (Area under *Striga* Number Progressive Curve) of wild and cultivated sorghum accessions across three field sites.

| **Area Under *Striga* Number Progressive Curve (AUSNPC)** | | | | | | | | | | | | |
| --- | --- | --- | --- | --- | --- | --- | --- | --- | --- | --- | --- | --- |
| **Bukedea** | | | | | **Kumi** | | | | **Alupe** | | | |
|  | **Day 44** | **Day 58** | **Day 76** | **Day 86** | **Day 44** | **Day 58** | **Day 76** | **Day 86** | **Day 44** | **Day 58** | **Day 76** | **Day 86** |
| **WSE-1** | 5.83±2.81^g^ | 91±28.74^h^ | 271.83±62.86^h^ | 481.83±57.19^d^ | 0.0±0.0^g^ | 22.17±4.58^h^ | 106.17±16.30^g^ | 304.5±35.22^c^ | 0±0^g^ | 25.66±2.33^h^ | 107.3±8.41^g^ | 317.3±68.86^b^ |
| **WSA-1** | 71.17±13.58^c^ | 254.33±36.41^d^ | 494.67±53.73^e^ | 693±54.07^c^ | 25.67±7.15^e^ | 162.17±28.08^f^ | 471.33±56.19^d^ | 716.33±52.49^b^ | 19.6±6.79^d^ | 155.4±25.56^d^ | 336±53.85^d^ | 406±69.04^b^ |
| **WSA-2** | 10.5±4.69^f^ | 56±11.99^i^ | 186.67±21.81^i^ | 308±33.72^d^ | 0±0^g^ | 59.5±11.25^g^ | 201.83±35.57^e^ | 316.17±54.32^c^ | 7±3.83^f^ | 15.4±7.79^i^ | 35±6.64^h^ | 63±8.57^d^ |
| **WSD-2** | 18.67±9.51^ef^ | 144.67±21.28^f^ | 403.67±35.63^f^ | 703.5±47.50^c^ | 16.33±8.41^f^ | 227.5±46.03^d^ | 595±55.53^c^ | 782.83±61.96^b^ | 12.6±3.43^e^ | 67.2±18.59^f^ | 138.6±42.77^f^ | 224±60.37^c^ |
| **WSD-3** | 45.5±14.43^d^ | 218.17±26.95^e^ | 549.5±39.55^d^ | 810.83±42.21^bc^ | 32.67±7.59^d^ | 198.33±19.18^e^ | 477.17±35.29^d^ | 718.67±39.87^b^ | 8.17±2.81^f^ | 54.83±6.62^g^ | 117.83±9.11^g^ | 163.33±21.96^c^ |
| **WSA-3** | 152.83±22.24^a^ | 458.5±41.12^a^ | 722.17±46.05^b^ | 886.67±35.81^bc^ | 119±29.80^b^ | 383.83±59.49^b^ | 663.83±37.58^b^ | 879.67±33.40^ab^ | 37.33±11.38^b^ | 129.5±27.75^e^ | 227.5±36.22^e^ | 268.3±41.63^bc^ |
| **WSD-1** | 98±19.21^b^ | 372.17±34.88^b^ | 693±36.77^c^ | 928.67±25.15^b^ | 51.33±14.87^c^ | 289.33±46.87^c^ | 696.5±46.07^a^ | 949.67±28.84^ab^ | 56±14.51^a^ | 280±15.17^a^ | 585.2±21.64^c^ | 754.6±32.03^b^ |
| **N13** | 22.17±11.77^e^ | 112±19.96^g^ | 306.83±42.52^g^ | 476±70^d^ | 0.±0^g^ | 25.67±9.84^h^ | 121.33±21.51^f^ | 288.17±29.11^c^ | 29.17±17.37^c^ | 204.17±114.33^b^ | 712.83±253.1^a^ | 978.83±308.36^ab^ |
| **Ochuti** | 47.83±8.93^d^ | 344.17±95.47^c^ | 872.2±111.61^a^ | 1045.8±62.97^a^ | 270.67±120.18^a^ | 606±43.50^a^ | 78.67±14.87^h^ | 1095.75±62.21^a^ | 18.2±7.86^d^ | 171.5±42.76^c^ | 633.5±154.88^b^ | 1639.75±92.97^a^ |

Values are means ±SD under *Striga* infestation. Means on each column followed by the same letter are not significantly different (*p≤0.05*)
